# Musculoskeletal injuries sustained at the California, USA: Baja California, Mexico border

**DOI:** 10.1186/s40621-022-00392-8

**Published:** 2022-08-25

**Authors:** Kathryn D. Dwight, William T. Kent, Jan M. Hughes-Austin

**Affiliations:** Department of Orthopaedic Surgery, University of California, San Diego, 9500 Gilman Drive, Mail Code 0863, La Jolla, CA 92093 USA

**Keywords:** Musculoskeletal injuries, Border fence, Trauma

## Abstract

**Background:**

Individuals attempting to enter the USA from Mexico at non-authorized points along the border fence often sustain injuries requiring medical intervention. We evaluated characteristics of this patient population and their hospital care to better understand patient treatment needs. Given the high-velocity nature of these injuries, we hypothesized that higher pain scores would be associated with longer lengths of hospital stay.

**Methods:**

In this cross-sectional study, we selected records of all patients from 2013 to 2019 who received care by the Orthopaedic Surgery department following an injury sustained at the California-Baja California border. We evaluated demographics, musculoskeletal injuries, procedures, length of hospital stay (LOS), follow-up, and pain scores via retrospective chart review. We used linear regression, adjusting for age and gender, to evaluate associations between pain scores and hospital LOS.

**Results:**

Among all 168 patients, there were 248 total injuries comprised of 46% lower extremity, 15% upper extremity, 17% spine, and 4% pelvic injuries. Average age at injury was 33 ± 10, 74% were male, and 85% identified as Hispanic. Of this patient population, 68% underwent operative interventions, 26% sustained open injuries, and 21% required external fixation for initial injury stabilization. Thirteen percent were seen for follow-up after discharge. Spine (n = 42), pilon (n = 36), and calcaneus fractures (n = 25) were the three most common injury types. Average LOS for all patients was 7.8 ± 8.1 days. Pain scores were not significantly associated with LOS (* p* =  0.08). However, for every surgical procedure performed, hospital LOS was increased by 5.16 ± 0.47 days (*p* < 0.001).

**Conclusion:**

Many injuries incurred by patients crossing the border were severe, requiring multiple surgical interventions and a prolonged LOS. The higher number of procedures was significantly associated with longer LOS in all operatively treated patients. Future studies are needed to determine how we can optimize care for this unique patient population and facilitate post-discharge care.

## Introduction

The southwestern USA shares 1993 miles of border with Mexico (Department_of_Homeland_Security. 2018). Six hundred and fifty-four miles of this border is separated by a variety of vehicle and pedestrian barriers (Department_of_Homeland_Security. 2018). Over the years, there have been modifications to the fencing barrier, and heights of the blockade range anywhere from 3-foot vehicle barriers to 8- to 18-foot steel fencing pedestrian barriers. (Walls and Work 2018) More recently, these barriers have increased in height to 30 feet in some locations (Department_of_Homeland_Security 2020). Resulting from laws that made entering the USA without authorization a criminal misdemeanor, these barriers are designed to prevent border crossings between the USA and Mexico at any location besides designated immigration checkpoints (U.S.C. 1929, Goodson 2019).

Historically, people who crossed the border from Mexico were single men looking for work (Goodson 2019). Recently, however, those who cross include not only men, but also families or children escaping violent crime, unrestrained gangs, and failing economies in their home countries (Goodson 2019; DeLuca et al. 2010). Many individuals and families seek asylum, but the process of seeking asylum is very difficult and the proportion of claims granted are low, e.g., 13.7% in 2017 and 12.3% in 2018 (Goodson 2019; Department_of_Justice_Executive_Office_for_Immigration_Review 2019). Thus, individuals denied asylum as well as those who are denied screening and application for entry may attempt entry via other methods such as scaling the border fence, tunneling under the fence, or cutting through the fence (Walls and Work 2018; Goodson 2019). While injury may occur on the fence or when falling from height, exposure to severe heat and cold in the desert could also result in the need for urgent medical care in the USA. Given the proximity of San Diego, California, to the US–Mexico border, many people who are injured crossing the border at this location are treated at our institution—a level one trauma center.

Falls from height, such as those from the border fence, frequently lead to orthopedic injuries. Injury patterns vary and depend on fall height, accidental versus non-accidental fall pattern, mechanism of landing, and landing surface (Con et al. 2014; Papadakis et al. 2020; Rowbotham et al. 2019). Prior work by Burk and Palacio showed that in El Paso and the Rio Grande Valley, Texas, the injuries experienced by individuals crossing the US–Mexico border were among mostly men and predominantly involved the spine and lower extremity (Burk et al. 2017; Palacio et al. 2021). Consistent with this work, Koleski et al. have shown that musculoskeletal injuries are the primary reason for medical attention following crossing the US–Mexico border (Koleski et al. 2019); and in San Diego, the number of patients treated for injuries while crossing the border continue to increase, with the most recent increase in border wall height associated with higher numbers of deaths, injury severity scores, and burden of complex injured patients (Kelada et al. 2010; Liepert et al. 2022).

In addition to assessing patient demographics and injury patterns in our population like these prior studies, we also sought to evaluate treatments for injuries and resulting length of hospital stay as well as average and postoperative pain to better understand patients’ needs and facilitate care. We hypothesized that among patients who underwent surgery their pain would be associated with longer lengths of stay.

## Methods

### Study population

Patients who present to the UC San Diego Health System for medical care from the US–Mexico border are assigned “Immigration Health” insurance. Using this designation, we searched all patient records with “Immigration Health” insurance between October 31, 2013 (when the electronic medical record was established at our institution), and December 31, 2019. There were 443 patients with “Immigration Health” insurance who were treated by healthcare providers at UC San Diego during this period. Through chart review, we determined that of these 443 patients, 168 were treated by the Orthopaedic Surgery Department and this subset of patients were included for analysis. This study was approved by the University of California, San Diego (UC San Diego), Human Research Protection Program.

### Patient characteristics

Demographic, injury, procedure, hospital stay, and pain data were collected for each patient via retrospective chart review. Specifically, we assessed age at the time of injury, gender [male, female], ethnicity [Hispanic/Latino, non-Hispanic, Unknown], race [American Indian/Alaskan Native, Asian, Black/African American, Native Hawaiian/other Pacific Islander, other/mixed race, unknown, White], primary spoken language, injury severity score (ISS), Abbreviated Injury Scale (AIS) [head/neck, face, chest, abdomen/pelvis, extremities, and external], number and type of injury, number and type of surgical procedures, date of admission and discharge, procedure dates, pain scores, and whether patients returned for follow-up.

### Injuries, procedures, and hospitalization

Procedure and injury information were collected from consult notes and operative notes. Type and total number of injuries were identified and categorized by upper extremity [scapula, clavicle, humerus, forearm, or hand] lower extremity [femur and any injury distal to femur], spine [cervical, thoracic, or lumbar spine], or pelvic [pelvic ring, acetabulum, or sacrum] injuries. If there was no bony involvement, injuries were classified as soft tissue injuries. Injuries were also defined as open or closed. Procedures were defined as each surgical intervention per injury and included external fixation application, open reduction internal fixation of the fracture/dislocation, and incision and debridement of wounds/fractures.

Length of hospital stay was defined and analyzed as days from admission to surgery and days from surgery to discharge. Pain scores were measured using the visual analogue scale (VAS) with scores from 1 to 10, and collected by a nurse each time vital signs were recorded, when pain medication was administered, and 30 min after intravenous pain medication or 60 min after oral pain medication was given. Pain scores were averaged over the patient’s length of stay and on postoperative day one (POD1) following surgical intervention. Additionally, the chart was reviewed for any postoperative follow-up appointments the patients attended.

### Statistical analysis

Descriptive analysis for this population included age, gender, ethnicity, race, language, ISS, AIS, and injury type. Patient characteristics were analyzed overall and by operative and non-operative treatment status. If patients had both operative and non-operative injuries, they were included in the operative treatment group. Patients were further subdivided and analyzed according to location of injury: upper extremity, lower extremity, spine, or pelvis. Patients were included in each category per anatomic location injured. If they had multiple injuries, they were included in the analysis for each.

Linear regression analysis using SPSS (version 27) was used to examine the associations of pain scores, number of procedures, and number of injuries with length of stay where *p* < 0.05 indicated statistical significance. Analyses were adjusted for age and gender.

## Results

We identified 168 patients assigned “Immigration Health” insurance who sustained a total of 248 musculoskeletal injuries. Characteristics for all patients as well as for patients stratified by operative and non-operative care can be found in Table 1. Average age of patients was 33.0 ± 9.7, 74.4% were male, 84.5% were of Hispanic ethnicity, 10% were White, 4% were Asian, and 78.6% of patients reported Spanish as the primary spoken language. The remaining patients spoke up to at least nine different additional languages. Average injury severity score (ISS) was 9.0 ± 6.5. Highest average Abbreviated Injury Scale (AIS) scores were for head/neck at 2.5 ± 1.0 and abdomen/pelvis at 2.5 ± 0.6. Thirty-five percent of patients experienced more than one injury. Patients had a range of one to seven injuries with all but one patient having between one and three injuries. Lower extremity injuries were the most common injury location occurring in 46% of patients.

**Table 1:** Characteristics of patients who were treated at UC San Diego after sustaining musculoskeletal injuries at the border between the USA and Mexico from 2013–2019

	All patients (n = 168)	Operative care (n = 115)	Non-operative care (n = 53)
Age, mean (SD)	33.0 (9.7)	32.9 (9.8)	33.1 (9.4)
Gender, % Female	25.6	24.3	28.3
*Ethnicity, %*			
Hispanic	84.5	84.4	84.9
Non-Hispanic	14.3	14.7	13.2
Unknown	1.2	0.9	1.9
*Race, %*			
Unknown/no record	4.8	6.1	1.9
Other	81.5	82.6	79.3
White	10.1	8.7	13.2
Asian	3.6	2.6	5.7
*Primary language, %*			
Chinese	0.6	0.9	–
English	9.5	7.8	13.2
Guajarati	0.6	0.9	–
Hindi	1.8	2.6	–
Mandarin	2.4	1.7	3.8
Other	2.4	1.7	3.8
Punjabi	2.4	2.6	1.9
Russian	0.6	–	1.9
Spanish	78.6	80.0	75.5
Unknown	1.2	1.7	–
Injury severity score, mean (SD)	9.0 (6.5)	8.5 (5.4)	10.2 (8.3)
*Abbreviated Injury Scale, mean (SD)*			
Head/neck	2.5 (1.0)	2.3 (0.6)	2.7 (1.2)
Face	1.8 (0.7)	1.7 (0.2)	1.8 (0.4)
Chest	2.4 (0.7)	2.5 (0.8)	2.3 (0.6)
Abdomen/pelvis	2.5 (0.6)	2.7 (0.6)	2.3 (0.6)
Extremities	2.3 (1.0)	2.4 (1.1)	1.9 (0.6)
External	1.0 (0.3)	1.1 (0.3)	1.1 (0.3)
Number of injuries, mean (SD)	1.5 (0.8)	1.5 (0.8)	1.4 (0.6)
*Multiple injuries, %*			
Bilateral	14.9	18.3	7.5
Unilateral	8.3	9.6	5.7
*Number of injuries, %*			
One	65.5	63.5	69.8
Two	32.2	23.5	22.6
Three	10.7	12.2	7.5
> Three	0.6	0.9	–

Table 2 presents the number and percentage of injuries by fracture type or soft tissue injury. The three most common types of fractures were spine (17%), pilon (14%), and calcaneus (10%). The most common type of soft tissue injury was a laceration (2.4%).

**Table 2:** Musculoskeletal injuries sustained by patients treated at UC San Diego after crossing the border between the USA and Mexico from 2013 to 2019

Injury	n (%)
*Fracture* Spine Pilon Calc Ankle Talus Foot Pelvis Plateau Tibia Hand Distal radius Humerus Fingertip amputation Forearm Patella Hip Femur Scapula Clavicle Fibula	42 (16.9) 36 (13.5) 25 (10.0) 18 (7.2) 16 (6.4) 11 (4.4) 11 (4.4) 11 (4.4) 11 (4.4) 8 (3.2) 7 (2.8) 6 (2.4) 5 (2.0) 5 (2.0) 5 (2.0) 4 (1.6) 3 (1.2) 3 (1.2) 2 (0.8) 1 (0.4)
*Soft tissue injury* Laceration Tendon Traumatic Arthrotomy Compartment Syndrome Nerve Sprain	6 (2.4) 6 (2.4) 2 (0.8) 2 (0.8) 1 (0.4) 1 (0.4)
Total	248

Table 3 shows the number and percentage of all, operatively treated, and non-operatively treated patients with an open injury, external fixator placement, and follow-up appointments attended. In particular, 26% of patients experienced an open injury, and 21% underwent external fixator placement. External fixators were in place for an average of 11.27 ± 6.8 days. Approximately 32% of lower extremity injuries required external fixation, and 62% of upper extremity injuries were open. Thirteen percent of all patients returned for follow-up after discharge, and among patients who underwent surgical intervention, 18% returned for follow-up.

**Table 3:** Injury details by operative (Op) or non-operative (Non) care and injury site for patients who were treated at UC San Diego from 2013–2019 after sustaining musculoskeletal injuries at the US–Mexico border

	All patients			Lower extremity			Upper extremity			Spine			Pelvis		
	All	Op	Non	All	Op	Non	All	Op	Non	All	Op	Non	All	Op	Non
	n = 168	n = 115	n = 53	n = 114	n = 91	n = 21	n = 37	n = 15	n = 18	n = 42	n = 11	n = 24	n = 10	n = 5	n = 3
Open Injuries n (%)	34 (25.6)	30 (26.1)	13 (24.5)	27 (23.7)	22 (24.2)	4 (19)	23 (62.1)	10 (66.6)	11 (61.1)	4 (9.5)	1 (9.1)	1 (4.2)	2 (20)	2 (40)	0 (0)
Required ex-fix^a^, n (%)	36 (21.4)	36 (31.4)	0 (0)	36 (31.6)	36 (39.6)	0 (0)	1 (2.7)	1 (6.7)	0 (0)	2 (4.8)	0 (0)	0 (0)	0 (0)	0 (0)	0 (0)
Post-op follow-up, n (%)	22 (13.1)	21 (18.3)	1 (1.9)	19 (16.6)	17 (18.7)	2 (8.7)	3 (8.1)	2 (13.3)	0 (0)	1 (2.4)	0 (0)	0 (0)	1 (10)	1 (20)	0 (0)

^a^External fixator

Table 4 demonstrates injuries, procedures, length of stay, and pain for all patients, for patients by extremity and for patients subcategorized by operative and non-operative treatment. Patients underwent 0–8 procedures to treat their injuries. Average number of injuries per patient was 1.5 ± 0.8, and patients with pelvic injuries had the highest average number of injuries with 2.1 ± 0.7. However, patients with operative lower extremity injuries had the highest average number of procedures (2.0 ± 1.3), and longest average length of stay (10.7 ± 8.8 days). In this patient group, the number of procedures consisted of external fixator placement followed by surgical stabilization(s). Patients with operative lower extremity injuries also had the highest average pain scores and POD1 pain scores with 4.6 ± 1.8 and 5.5 ± 2.1, respectively. Non-operative lower extremity injuries and non-operative spine injuries had the shortest average length of stay at 3.7 ± 5.2 days and 3.8 ± 5.3 days, respectively.

**Table 4:** Injury, procedure, length of stay, and pain characteristics^a^ by operative (Op) and non-operative (Non) care and injury site for patients who were treated at UC San Diego from 2013–2019 after sustaining musculoskeletal injuries at the US–Mexico border

	All			Lower extremity			Upper extremity			Spine			Pelvis		
	All	Op	Non	All	Op	Non	All	Op	Non	All	Op	Non	All	Op	Non
	n = 168	n = 115	n = 53	n = 114	n = 91	n = 21	n = 37	n = 15	n = 18	n = 42	n = 11	n = 24	n = 10	n = 5	n = 3
Number of Injuries	1.5 (0.8)	1.5 (0.9)	1.4 (0.6)	1.6 (0.9)	1.5 (0.9)	1.6 (0.7)	1.8 (0.8)	1.8 (0.9)	1.6 (0.7)	1.7 (1.1)	1.9 (1.8)	1.5 (0.7)	2.1 (0.7)	2.4 (0.9)	1.7 (0.6)
Number of Procedures	1.1 (1.1)	1.8 (1.2)		1.6 (1.4)	2.0 (1.3)		0.7 (0.8)	1.3 (0.6)		0.6 (0.9)	1.5 (1.0)		1.1 (1.0)	1.8 (0.8)	
*Length of stay, days*															
Total	7.8 (8.1)	9.7 (8.4)	3.9 (6.0)	9.3 (8.7)	10.7 (8.8)	3.7 (5.2)	5.7 (7.4)	5.1 (5.2)	6.2 (9.5)	6.1 (5.9)	9.6 (5.3)	3.8 (5.3)	8.6 (6.0)	7.8 (2.4)	10.0 (12.1)
Admission to Surgery	1.1 (1.5)	1.1 (1.5)		1.1 (1.5)	1.1 (1.5)		1.0 (1.3)	1.1 (1.3)		1.9 (2.4)	1.8 (2.3)		1.6 (1.3)	1.2 (1.3)	
Surgery to discharge	4.3 (4.6)	4.3 (4.6)		4.3 (4.9)	4.4 (4.9)		3.2 (2.6)	2.9 (2.7)		5.6 (3.7)	6.7 (3.9)		5.9 (2.4)	5.8 (2.9)	
*Average pain, 1–10*															
Hospital stay	4.1 (2.0)	4.4 (1.8)	3.5 (2.2)	4.2 (1.9)	4.6 (1.8)	2.9 (1.8)	4.0 (2.2)	3.5 (1.7)	4.5 (2.7)	3.6 (1.7)	4.4 (1.0)	3.2 (1.9)	3.9 (1.4)	3.6 (1.0)	3.8 (0.7)
Post-op day 1	4.9 (2.2)	4.9 (1.3)		5.9 (2.1)	5.5 (2.1)		4.2 (2.8)	4.0 (3.0)		4.9 (1.8)	4.7 (1.8)		3.7 (2.3)	3.7 (0.4)	

^a^mean (SD)

As shown in Table 5, there was no association between average overall pain score or POD1 pain score and LOS when comparing all operatively treated patients in unadjusted analysis (*p* = 0.08) or analysis accounting for age and gender (*p* = 0.11). This is consistent with similar analysis in patients with upper and lower extremity injuries. There was also no association between number of injuries and LOS in any group analyzed. There was a significant association between the number of procedures and LOS in all operatively treated patients (*p* < 0.001). Adjusted for age and gender, for each procedure performed, patients had an additional 5.16 days longer stay in the hospital (*p* < 0.001). This association was also significant in the upper and lower extremity sub-analyses and in patients with operative upper extremity injuries. Specifically, adjusted for age and gender, these patients had 7.71 days longer stay in the hospital per procedure performed (*p* = 0.01).

**Table 5:** Association of pain scores, number of injuries, and number of procedures with hospital length of stay (LOS) among patients who were treated at UC San Diego from 2013–2019 after sustaining musculoskeletal injuries at the US–Mexico border

	Pain and LOS		POD1 Pain and LOS		No. injuries and LOS		No. procedures and LOS	
	B (SD)	p-value	B (SD)	p-value	B (SD)	p-value	B (SD)	p-value
*All*								
Unadjusted	0.57 (0.32)	0.08	–	–	0.96 (0.76)	0.21	–	–
Age and gender adjusted	0.52 (0.32)	0.11	–	–	1.04 (0.75)	0.17	–	–
*Operative*								
Unadjusted	0.74 (0.44)	0.09	0.65 (0.37)	0.08	−0.18 (0.90)	0.85	**5.20 (0.47)**	** < 0.001**
Age and gender adjusted	0.62 (0.44)	0.16	0.60 (0.38)	0.11	−0.01 (0.90)	0.99	**5.16 (0.47)**	** < 0.001**
*Lower extremity*								
Unadjusted	0.51 (0.52)	0.33	0.79 (0.45)	0.08	−0.25 (1.04)	0.81	**5.21 (0.51)**	** < 0.001**
Age and gender adjusted	0.41 (0.52)	0.44	0.74 (0.45)	0.10	−0.13 (1.05)	0.90	**5.17 (0.51)**	** < 0.001**
*Upper extremity*								
Unadjusted	0.54 (0.84)	0.53	0.17 (0.49)	0.74	2.71 (1.50)	0.09	**6.13 (1.62)**	** < 0.001**
Age and gender adjusted	0.30 (0.79)	0.72	0.31 (0.51)	0.49	1.40 (1.76)	0.44	**7.71 (2.40)**	**0.01**

Bold values indicate statistical significance

Statistical significance at *p* < 0.05. POD1 = Postoperative Day 1

## Discussion

From 2013 to 2019, patients who received care from Orthopaedic Surgery at our institution experienced a variety of musculoskeletal injuries while attempting to cross non-authorized points along the border from Mexico into the USA. Most of the patients were young, Hispanic men. However, the multitude of different primary languages spoken demonstrates diversity within this population. Lower extremity injuries were the most common injury type resulting in the highest number of procedures with highest average pain scores and longest lengths of stay. Thirty-five percent of patients experienced multiple injuries, and approximately one-fifth of all injuries were severe enough to require initial stabilization with external fixators. We found no association between either average pain scores throughout hospitalization or POD1 pain scores with hospital LOS. Instead, we found that the number of operative procedures was significantly associated with a longer hospital length of stay in all operatively treated patients. Despite Orthopaedic Surgery being involved in the care of all patients, only 13% of all patients were seen in clinic for follow-up.

Among our patient population, lower extremity injuries were the most common injury type (46%) which is consistent with prior studies conducted at the Texas–Mexico border, and lower extremity injuries occurred 58–60.7% of the time (Burk et al. 2017; Palacio et al. 2021). In our population, lower extremity injuries had an average of 1.6 ± 1.4 procedures, including application of external fixators for stabilization of the injury.

This patient population can arrive severely injured, requiring significant time and effort on the part of the surgeon to ensure appropriate care. Approximately 25% of injuries were open which demonstrates the injury severity in this population. This contrasts with the general population, in which approximately 1.9–3.6% of fractures per year are defined as open (Court-Brown et al. 2015). The most common mechanism of injury for open fractures was crush injuries followed by falls from height (Court-Brown et al. 2015). Patients who sustain high-energy mechanisms of injury (i.e., falls from height) have a higher likelihood of open fractures compared to the average population. Therefore, given the higher severity of injury, we initially hypothesized that higher pain scores would be associated with longer lengths of hospital stay. While we did not find an association between higher pain scores and longer lengths of stay, we found that a higher number of operative procedures were associated with a longer hospital stay. Our patients had an average LOS of 9.3 ± 8.7 days which is four days longer than the average nationwide LOS for musculoskeletal injuries which was reported at 5.4 days in 2011 (Pollack et al. 2014). Given the severity of injury requiring multiple procedures, our findings suggest that in-hospital care planning should assume prolonged LOS in this patient population. By preemptively gauging the likely number of procedures each patient would require based on the type and mechanism of injury, we can better determine hospital LOS and help prepare postoperative discharge planning.

Approximately 18% of operatively treated patients were seen for a postoperative follow-up examination. Once discharged, most patients are unable to return to the USA for their follow-up care. The ones who were able to return did so because they were in the process for asylum within the USA and were temporarily housed at Immigration and Customs Enforcement (ICE) detention centers, which afforded them the opportunity to return for an examination (US_Immigration_and_Customs_Enforcement 2020). Given this barrier to follow-up care by our surgeons, we are unable to address any postoperative complications. Thus, postoperative follow-up care depends on the patients seeking care once they return home. This limitation presents an opportunity to address postoperative care for patients along the California–Mexico border.

Our investigation spans a 7-year period (2013–2019) prior to the change in border wall height that occurred in 2020, so it does not include injuries associated with the higher wall. It includes, however, periods of policy changes, e.g., zero tolerance and metering, affecting individuals seeking asylum at the southwest USA border. Liepert and colleagues observed a surge in injuries and injured persons, even with a taller border wall, in response to Title 42, a COVID-19-related policy stating migrants can be expelled by Customs and Border Protection without asylum screening (Liepert et al. 2022). These findings, along with others and ours, suggest that given the limited opportunity for asylum, many people will continue their pursuit of entry into the USA at non-authorized points in order to flee violence, unrestricted gangs, and failing economies, and some will need surgical care. While we depend on changes in policy to prevent trauma at the US–Mexico border, we have the opportunity to address postoperative care and discharge planning to facilitate a successful outcome and lessen the need for an extended hospital stay.

While our study has many strengths, it also has several limitations. First, UC San Diego Health System splits border call with Scripps Mercy hospital in San Diego. Therefore, this subset of patients is approximately half of the patients who are injured at the border near San Diego. Although this limits our sample size, there is likely no bias as this shared border call rotates every other month throughout the year. Second, polytrauma patients requiring intubation were unable to provide pain scoring while intubated. Therefore, the pain scores for these patients only include pain data for when the patients are awake and conversant, and therefore able to rate their pain on a scale of one to ten. Third, some patients with more minor injuries, i.e., patients with isolated hand injuries, were able to be discharged on the day of surgery, POD0, and therefore do not have POD1 pain score data to include in the analysis. Fourth, while we minimized barriers to communication using My Accessible Real-Time Trusted Interpreter (MARTTI) video interpreters, there could have been miscommunication in the self-report of pain. Further, we could not account for any other motivations to underreport pain or avoid analgesia, which could have led to lower reported pain scores. Pain reporting and pain management in this patient population warrant further study. Comparisons were made between LOS and pain scores which showed no association; however, this is limited by a small sample size which could have missed a potential association.

Strengths of this study include the investigation of care and pain in this patient population, which has not been explored previously. Given that the data were analyzed via direct chart review, we did not rely on procedure coding measures to define patient injuries and we were able to include non-operative injuries in our analysis. Thus, we were able to better capture more subtle aspects of musculoskeletal injuries in this cohort.

## Conclusion

In conclusion, this patient population consists of mostly young, Hispanic, men who sustained severe, mostly lower extremity injuries requiring a lengthy hospital stay, and frequently, multiple procedures. Longer hospital lengths of stay were significantly associated with higher number of procedures, but not with reported pain scores. Postoperative follow-up care is limited in this patient population and warrants additional research to determine best practices for successful outcomes once discharged.

## Data Availability

The data that support the findings of this study are available on request from the corresponding author [JHA]. The data are not publicly available due to containing information that could compromise research participant privacy/consent.

## Abbreviations

LOS: Length of stay
POD1: Postoperative day 1
ICE: Immigration and customs enforcement
